# School-based learning about sugary drinks: possibilities and potential for curriculum approaches supporting health promotion in New Zealand

**DOI:** 10.1093/her/cyae020

**Published:** 2024-06-19

**Authors:** Suzanne Trask, Simon Thornley, Gerhard Sundborn

**Affiliations:** Liggins Institute, University of Auckland, 85 Park Road, Grafton, Auckland 1023, New Zealand; School of Population Health, Faculty of Medical and Health Sciences, University of Auckland, 22-30 Park Ave, Grafton, Auckland 1023, New Zealand; School of Population Health, Faculty of Medical and Health Sciences, University of Auckland, 22-30 Park Ave, Grafton, Auckland 1023, New Zealand

## Abstract

Achieving greater alignment with national curriculum and local school and teacher objectives alongside a deeper understanding of student needs can enhance the impact and reach of health promotion interventions. This study reports on teacher perspectives of a multi-pathway curriculum outline supporting learning (Grades 7-9) about sugary drinks. The outline was developed to support scale-up and sustainability of a successful sugary drink intervention trialed in four New Zealand secondary schools. Sixteen teachers from a range of subjects provided input via focus groups. Inductive qualitative thematic analysis was used to identify and interpret patterns within the data. Sugary drinks were perceived to be an important and engaging learning context. Teachers valued the potential long-term societal benefits of health-based learning and benefits to individual students and their families. They recognised students as health communicators and influencers within families and communities. Relevance to students’ lives and alignment with national curriculum and assessment objectives and teacher subject expertise were key factors in learning pathway selection. Teacher support is crucial in facilitating sustainable school-based health promotion, which often does not sit within a single curriculum area. Factors such as these, that teachers prioritise in their curriculum decision-making, must be understood and leveraged in school-based health promotion research.

## Introduction

Sugary drinks, also known as sugar-sweetened beverages, refer to any beverage containing added sugar or other sweeteners, including soda (fizzy drink or soft drink), fruit drinks and sports drinks [1]. They are a major source of added sugars but are not a necessary part of a healthy diet. Sugary drinks are nutrient-poor and high in concentrated fructose, and there are many better beverage alternatives. Sugar in liquid form (beverages) is more detrimental to metabolic health than sugar in solid form (in foods) as the sugar is more quickly ingested and readily absorbed [1–4]. Overconsumption of sugary drinks is directly associated with weight gain and increased risk of developing noncommunicable diseases including Type 2 diabetes and dental caries [4, 5]. Products with high sugar content impair concentration, learning and behaviour [2]. Moreover, high sugary drink intake frequently clusters with other unhealthy lifestyle behaviours [6]. However, as with other nutritional and substance consumption behaviours, drivers of the purchase and consumption of sugary drinks are complex.

School-based education is a key component in health promotion to increase self-efficacy towards healthy behaviours for individuals and society. These programmes are effective in changing attitudes and behaviour relating to sugary drinks [7, 8]. Teachers are key influencers in school-based health promotion, and therefore it is important to understand the factors that they prioritize in their curriculum decision-making [9]. In this study, we report on teacher perspectives of a multipathway curriculum outline supporting school-based learning (Grades 7–9) about sugary drinks. This research was conducted as part of the New Zealand FIZZ Project, where researchers work with schools and communities from low socioeconomic areas/with high Māori and Pacific populations and the government to advocate for action to reduce intake of sugary drinks [8, 10] (https://fizz.org.nz).

In New Zealand, a 2020–21 Ministry of Health Survey revealed an increase in childhood obesity rates, with approximately one in eight children (12.7%) (aged 2–14 years) classified as obese compared to 1 in 10 the previous year. Obesity prevalence varied by ethnicity, with higher rates among Māori (17.8%) and Pacific children (35.3%). Socioeconomically deprived areas showed a disproportionately higher incidence of childhood obesity, with children being 2.5 times more likely to be obese compared to those in less deprived areas [11]. While New Zealand households have made small positive shifts in beverage choices, addressing sugar-sweetened beverage consumption remains crucial for public health [12].

A previous FIZZ intervention drawing on social marketing approaches and gamification to increase knowledge of sugar content in beverages and health risks associated with excess sugar intake was found to be effective in changing attitudes towards the overconsumption of sugary drinks in the short term [8, 10]. The curriculum outline discussed in this study was developed to support the scale-up and longer-term sustainability of this intervention and extend student learning about complex influences driving the availability and overconsumption of sugary drinks.

## School-based health promotion

Education for health promotion can support the development of health literacy; that is, personal knowledge and competencies that enhance the readiness and ability to access and effectively use information to improve individual and community health [13, 14]. Knowledge developed from exploring trustworthy evidence can potentially increase perception of risk and influence attitudes and beliefs, enhancing motivation for change [15, 16]. A key window for health promotion education is the adolescent life stage (10–18 years), characterized by increasing independence and separation from parental influence [17]. The school environment, where adolescents spend a large proportion of their time, is thus an important setting for this education. Therefore, creatively integrating health learning into core curriculum efforts at this age and stage becomes critical, as this can address health and education outcomes. Drawing on the perspectives, experiences and expertise of partners from both sectors is vital, especially given the constraints of limited time, crowded school curricula and the need for leadership and oversight within schools of health research initiatives [18, 19]. Who drives these initiatives and the methods employed are key considerations, alongside establishing measures for evaluating effectiveness that are feasible and not burdensome for teachers or students [20, 21].

In many education systems around the world, including New Zealand, the national curriculum grants schools and teachers local authority to design learning that is suited to the needs and interests of their students. [22–24]. If teachers perceive materials to be relevant to their learners and aligned with national and local curriculum objectives, they are more likely to use them; if not, the risk of nonimplementation and subsequent drop-off increases, negatively impacting programme sustainability [25]. Materials must be designed and structured to support teacher adaptation while respecting the integrity and intent of the health-based (and other) learning objectives [26, 27]. By focusing on teacher perspectives, this study adds to knowledge about the education-specific practicalities and relevance of school-based education for health promotion initiatives.

## Materials and methods

The FIZZ project is a long-term partnership between health research and four low socioeconomic status schools in New Zealand to promote behaviour change in the context of reduced sugary drink consumption [8, 10] (https://fizz.org.nz). The previous health-led FIZZ programme drew on popular culture of Pasifika secondary school students (aged 11–14 years from four co-educational high schools in the North Island of New Zealand). The schools have equity index (EQI) ratings between EQI500 and EQI525, meaning that these schools experience a high level of socioeconomic disadvantage that negatively impacts student achievement. The EQI rating is used to determine the level of equity funding each school receives. EQI numbers for New Zealand schools range from the least socioeconomic disadvantage 344 to 569 for the greatest socioeconomic disadvantage [28].

The FIZZ programme was well-supported by students and staff and showed promise as an educational intervention. There were significant learning gains in knowledge of the ‘3–6–9’ teaspoon rule for daily sugar intake and the amount of sugar in commonly purchased drinks. Teachers who were involved in the original FIZZ intervention commented that it was successful because it achieved visibility and influence in the schools. A classroom learning programme was augmented by visible signage, concert events led by a well-known rap artist and competitions with desirable prizes such as Fitbit smart watches, sports apparel (t-shirts, caps), family movie tickets, digital headphones and speakers, a playstation 4 console and branded drink bottles. According to teachers, this prominent presence encouraged discussions and awareness about the importance of reducing sugary drink intake. However, the intervention model was unsustainable for reasons including resourcing and time constraints, from within both school and health. The development of the curriculum outline discussed in this follow-up study was a postintervention response to the need for

- scale-up and sustainability of learning (i.e. the programme needed to be school-led) and
- a recognition of the challenge of supporting action on knowledge to achieve and sustain long-term behaviour change.

The present study sought to understand teacher perspectives and the challenges and opportunities teachers identified when partnering with health researchers to develop curricular learning to promote long-term health behaviour change in relation to sugary drink consumption. By focusing on teacher perspectives, this study gains insights into the practicalities of curriculum adaptation, the relevance of the intervention to their educational settings and the strategies they consider effective for sustaining and scaling up the learning gains achieved through the FIZZ project.

The research question was: What are teacher perspectives and priorities in curriculum decision-making related to health promotion education about sugary drinks?

Ethical approval was gained for the study from the University of Auckland Human Participants Ethics Committee on 20 April 2023 (Ref: UAHPEC25650). All four original intervention schools were invited to participate in this follow-up study. Three schools indicated their interest in participation. Teachers and senior leaders from these three schools were provided with the draft curriculum outline for review (Table I). They were then invited to attend a 1-h focus group to provide input on the materials. To reduce travel and time demands for teacher participation, one focus group was held at each of the schools. During the focus group, teachers were asked for their impressions of the learning outline and to offer suggestions for improvement. They were invited to share their thoughts on the role of school–health interactions and the level of detail and support required for teachers to integrate learning about sugary drinks into their planned curriculum.

**Table I.** Focus group participants

Focus group	Participant code	Role in school		Learning area expertise
School 1 (S1) Co-Educational Roll Band: 1500–1999	T1	Y	DP	Health and Physical Education (H&PE)
	T2		HoD	H&PE
	T3		Teacher	Food Technology (F&T)
	T4		Teacher	H&PE
	T5		HoD	Science (Sci)
	T6	Y	DP	Humanities (Hum)
School 2 (S2) Co-Educational Roll Band: 2000+	T1		Teacher	Biology and Horticulture (Bio&Hort)
	T2		Teacher	Science and Biology (Sci&Bio)
	T3		Teacher	Science and Biology
	T4		Teacher	Science
	T5		Teacher	Science and Chemistry (Sci&Chem)
	T6		Teacher	Science and Physics (Sci&Phys)
	T7		Teacher	Science and Chemistry
	T8	Y	Teacher	Science and Biology
School 3 (S3) Co-Educational Roll Band: 500–999	T1	Y	HoD	Science and Biology
	T2	Y	HoD	H&PE

Participant code: T = teacher; role in school = involvement in original FIZZ intervention (Y = yes); HoD = Head of Department; DP = Deputy Principal.

Audio recordings of focus groups were transcribed using intelligent transcription by the first author (S.Tr.), who led the focus groups with G.S. This ensured that understanding of the context and original conversations was maintained. Inductive descriptive thematic analysis of transcripts and other data sources including meeting notes and curriculum development drafts involved close reading and familiarization with data, interpreting and assigning individual data items to codes and aggregating codes to form themes to answer the research question. Initial interpretations by the first researcher were discussed with S.Th. and G.S. to reach consensus [29].

### The FIZZ curriculum outline

Learning foci included the following:

- the availability and overconsumption of sugary drinks as a health issue
- the science of the effect of sugar in the body
- complex influences on how we access and choose to consume sugary drinks
- the science of behaviour change
- systems-level barriers and enablers to behaviour change such as social, political, marketing and economics.

Learning was aligned with the New Zealand National Curriculum (NZC) Science and Health and Physical Education learning areas at Levels 5 and 6 (ref). To accommodate the possibility for learning in multiple curriculum areas such as science, health and physical education, food and nutrition, the outline provided teacher support in the form of pathways and ‘on-ramps’ [30] (p. 992) such as suggested learning objectives and activities drawing on pedagogies that were likely to engage diverse learners.

### Instructional strategies

The materials were designed by drawing on the following research-based strategies:

- The Va’atele Framework [31]: Si’ilata’s Va’atele is the Samoan name for the ocean-voyaging double-hulled canoe of Pasifika peoples. The Va’atele metaphor is used as a framework for Pasifika learners’ success to demonstrate how educators might privilege and utilize students’ linguistic and cultural resources within curriculum learning at school (p. 930–931). This included opportunities for students to present/write in their own language and use oral methods and group and collaborative tasks.

Social marketing and gamification strategies used in the original FIZZ intervention were incorporated and extended due to their proven effectiveness.

- Social marketing: Social marketing approaches recognize that behaviour is influenced by a wide range of factors including social norms, cultural values, personal beliefs and environmental influences [32, 33]. Social marketing uses traditional marketing approaches for the benefit of individuals and society, e.g. identifying and analysing complex underlying factors that contribute to sugary drink consumption and using that knowledge to raise awareness.
- Gamification: Gamified elements were incorporated to enhance student engagement and learning [34], e.g. competition, points, rewards, challenges, inclusion of choice and autonomy in learning, opportunity for collaboration and social interaction.

The outline and resources including images and video clips to stimulate classroom discussion and adaptable worksheets are available at https://fizz.org.nz.

## Results

As illustrated in Table II, a number of key themes were identified. An examination of these themes (highlighted in single quotes) and subthemes, supported by illustrative quotes, is presented later.

**Table II.** Summary of findings: teacher perspectives and priorities in curriculum decision-making related to health promotion education about sugary drinks

Theme	Subthemes
Teacher impressions of the FIZZ curriculum outline	Teacher responsibility to develop students’ interest in the topic
	Appreciation of the frameworks/strategies used
Teacher curricula and pedagogical decision-making	Importance of health and education collaborations
	Prioritization of national curriculum and assessment requirements
	Potential of FIZZ learning for integrated curriculum
	Prioritization of relevance to learners’ lives
	Importance of understanding of learner needs
	Importance of scope for teacher adaptation
Teacher recognition of complex influences on sugary drink consumption	Knowledge or lack thereof about sugary drinks
	Influences of parental and home environments
	Knowledge of sugar in relation to nutrition/physical/mental health
	Cost/convenience factors related to sugary drinks
	Marketing/economic factors related to sugary drinks
	Change over time as a factor in sugary drink consumption
Adolescent influence on health behaviours	Adolescents influencing decision-making in their homes
	Importance of strengthening school–home connections
Adolescent action on learning	Students understanding that they can make a contribution
	Suggestions for student actions and approaches
	Using evidence to inform decision-making and action
Teacher professional learning	Teacher desire to develop their own knowledge
	The need for teacher professional learning

### Teacher impressions of the FIZZ curriculum outline

Teachers universally agreed that sugary drinks were an important and engaging context, offering potential for the exploration of health and science issues. One respondent asserted that as teachers, they had a responsibility to the wider community to support health-oriented learning that improves lives.

I think the key thing is, that it’s authentic learning that they can apply to their lives, that it actually improves their lives. We actually owe it to our community to do that. (S1|T1|H&PE)

But as students might not naturally gravitate towards such learning, teachers play an important role in cultivating students’ interest and engagement in the topic. One teacher explained that a teacher’s role is to broaden students’ understanding and support them to see the relevance of the topic and possible health implications.

If you think of what a 15 year-old is interested in, it’s a very small package. So, if you ask the kids, nobody’s gonna say, sugary drinks and diabetes and obesity. (S3|T1|Sci&Bio)

This calls for a complex and nuanced understanding of students as individuals and learners and their interests, in relation to curriculum requirements. Teachers appreciated the connections to history and students’ cultures and the use of Si’ilata’s (2019) Va’atele Framework for Pasifika learner success.

*I love that your framework is a Pacific framework. I loved that at the beginning [there is] pre-learning, storytelling, what do you already know? What drinks historically does your culture have?* (S2|T8|Sci&Bio)

Teachers also noted that strategies for gamification would work well, supporting learning through enjoyable participation.

*Definitely the gamification is something that does work well with our kids. I mean, it’s about making it fun. If they see it as fun, then they’re more likely to participate then learn through just participating*. (S3|T2|H&PE)

### Teacher curricular and pedagogical decision-making:

Teachers agreed that the sugary drink context and outline suggested a range of learning pathways, depending on curriculum area and student interests. However, teachers emphasized the significance of codesign and partnerships between education and health sectors. One teacher encapsulated this argument, referring to appropriate levels of learning, student and school needs and the (ir)relevance of step-by-step ‘recipe-based’ lessons.

*Working with [scientists], if they don’t understand where our kids are at, they’re pitching at a level that’s not relevant to students and it just becomes a little bit pointless. I have been in situations like that, where we can’t get the researchers to basically understand what the needs in the school are, what the needs in the classroom are, and where the students are at. … I don’t want a recipe because the students here are different to the students down the road at a different school. So the whole recipe thing is not going to work. Whereas if you give suggestions, if you give ideas, teachers will take that. Step-by-step recipe lesson plans, I wouldn’t even look at them*. (S3|T1|Sci&Bio)

Teachers explained that they would select largely according to their own knowledge, pedagogical content knowledge (specialized knowledge enabling teachers to effectively teach specific content) [32], current curriculum specialities and interests. However, they saw scope and connections to other curriculum areas. For instance, a biology teacher explained her preference for locating learning about sugary drinks within biology and body systems, but saw value in making connections to other disciplines and curriculum areas.

I would go down the biology science body systems [track], because I’m a biologist. But I also see great scope for the chemistry of foods, and food label calculations which I think has value to the kids long term. I think they’re both really important. So the good thing about it is I think that there’s scope for multiple areas of the curriculum, not just health and science. (S2|T8|Sci&Bio)

A focus on alignment with national curriculum and high-stake assessment standards that teachers were required to deliver was a key consideration. Teachers framed and discussed their plans for learning about sugary drinks with reference to these, with support from researchers.

I’m interested to hear, especially as we get into the science standards, how [the sugary drink context] can roll into what we offer. (S1|T5|Sci)

We’ve just redesigned our level one standards, and I think there is scope for [the sugary drink context]. It could definitely fit in and be applied. [It] would be quite tricky, unless it was heavily supported by external documentation, like a program that yourselves [researchers] would run, which would then finalise within a new standard assessment. (S3|T2|H&PE)

I am actually really excited to be a part of this project because I’ve just recently come off of a level two assessment that is very connecting to [discussion of the sugary drink learning outline]. (S1|T4|H&PE)

Teacher prioritization of relevance to learners’ lives and learning impact was a central theme and one that was much-remarked upon. Teachers drew on their understanding of learner needs and local school communities to decide on their focus for learning.

I would group together the social change and body systems around disease because that’s the biggest impact a lot of our Pasifika families are having, heart disease and huge diabetes issues. (S3|T1|Sci&Bio)

A lot of the kids have family who have diabetes or something like that. So that’s very relevant to them. (S2|T4|Sci)

*A massive part of our curriculum area within health and physical education is connecting to some health related issues in the community*. (S1|T2|H&PE)

Teachers not only focused on immediate school-based goals but also recognized and valued potential long-term benefits of health learning for students and their communities. They recognized that by fostering knowledge and awareness, ‘adolescents can exert influence on decision-making processes and health behaviours’ in the home.

Generally Pacific families, it’s a very hierarchical kind of decision-making process. But encouraging kids that, this is a possibility, you can talk to older people about decisions that they make about the food. (S3|T1|Sci&Bio)

*[My teaching team] spoke about awareness, but not in a traditional campaign of posters, but really take it into home life. Not to criticize the families who are taking in heaps of sugar, but just to get the kids to be knowledgeable enough to explain to their families, the effects of the sugar*. (S3|T2|H&PE)

The strengthening of school–home discussions was identified as an important outcome and a pivotal opportunity to extend impact beyond school; however, it was recognized that achieving this influence is a challenge for which further support is needed.

At parent meetings I had three sets of parents talk to me about how [their children] were discussing diabetes with the wider family. One of the student’s grandma had diabetes, which the child knew, but the child didn’t know anything beyond that. And so mom was talking to me about how at the dinner table, they were discussing, what kind of diabetes do you have? What treatments do you have? And I thought that was amazing. And for me, that’s the kind of outcome that I would like to see. (S3|T1|Sci&Bio)

We want to try and see if students can influence their homes. And so that’s probably the biggest thing. And that’s where we are at the moment and trying to get to that place where we influence outside is kind of what we need assistance with. (S1|T2|H&PE)

Resources to support conversations in the home were identified as a potential area for focus.

If they know that we’re covering this with their children at school, then also you have resources that support the parent’s learning at home, perhaps to support a structured discussion with some family or somebody at home. (S1|T1|H&PE)

### Teacher recognition of complex influences on sugary drink consumption

Teachers drew on their broad general and specialist knowledge to recognize and discuss complex influences on what students are able to access and consume. They argued that education can and should increase knowledge and raise awareness of these complex factors, such as the impacts of sugary drink consumption on mental and physical health, along with other factors such as cost, convenience and taste. One teacher made reference to targeted marketing and numerous brands of sugary drinks. Another mentioned the significance of change over time, related to increased sugar intake.

How many of our kids can recognize sugary drink brands? How many do they know? Because that’s the marketing idea, it’s really aimed for children. (S2|T7|Sci&Chem)

If you think about diabetes and obesity, these weren’t [necessarily] issues 100 years ago, in the Pacific Islands and in New Zealand. (S3|T1|Sci&Bio)

Nutritional knowledge emerged as a factor for decision-making about learning for a food technology teacher.

The big gap is the lack of nutritional knowledge; why is this [beverage] so much better for you? (S1|T3|F&T)

Additional considerations, counter to the notion that students can influence decision-making in their homes, were cost factors and potential limitations on students’ abilities to influence parents’ purchasing decisions. In explaining this dynamic, one teacher inferred that there is potential for students to use their knowledge to facilitate future change.

*The kids don’t get to pick, they get whatever their parents are going to [buy]. And if it’s the cheapest, that’s what they’re getting. So it’s actually trying to teach them stuff that they might not be able to apply now, [but] it might be that they can apply it when they have the ability to apply it*. (S1|T1|H&PE)

Teachers emphasized the importance of ‘student action on learning’. This was seen as one way of fostering student awareness of their capacity to make a contribution and support change.

*The key idea is for the students to come up with an action* (S2|T5|Sci&Chem)

It was important for students to initiate and carry out action in a way that they experienced success, supporting them to ‘see’ themselves as being able to make a difference. One teacher suggested that this could help to increase confidence and address future apathy.

[It is important] for kids to understand that they have a role, and they can actually impact decision-making. Otherwise, you are developing young people that aren’t interested in voting, because of the ‘why bother’ apathy thing. (S3|T1|Sci&Bio)

Teachers suggested numerous approaches for student actions. Examples included using communication such as evidence-based argumentation or giving a community presentation. Teachers referred to a range of societal audiences: peers, school, community and corporate groups.

In science, we’re pushing [the use of] evidence. So another [idea] would be writing a prosecution document against Coca-Cola or …., like trying to collect the evidence as to why they should stop or change whatever it is that they’re doing. Because that means [students] have to integrate, re-sort information from different places. (S1|T5|Sci)

*If we could, for my class, I’d push for that Tuakana Teina (Te Reo Māori phrase meaning older sibling/peer-younger) type of relationship, like getting my Year 11 to teach Year 9. Maybe they could make a presentation. And even if we film it so that if they physically can’t be there to teach, we can show that video to our juniors*. (S2|T4|Sci)

I’m going to get [students] to [create] a dance. And then they can perform it in the school and then send the message. (S2|T1|Bio&Hort)

[Students] could write to the Principal, they could write to the Board of Trustees, they could even write to the local MPs (Members of Parliament) about reducing the number of sugary drinks available…so getting [students] to go the extra step in terms of the implementation of the action. (S1|T1|H&PE)

### Teacher professional learning

Teachers wanted to develop their own knowledge, and this was a motivation for joining with researchers. One teacher explained varied expertise as a reason why professional learning and development (PLD) was important for both conceptual and pedagogical knowledge in a context such as sugary drinks.

*There does need to be some [teacher] understanding of those big ideas. Because we have physics teachers and chemistry teachers, who probably don’t have that level of understanding. So I think PLD is good*. (S3|T1|Sci&Bio)

In summing up, these findings underscore the crucial role that teachers play in selecting and interpreting curriculum materials to facilitate learning that is relevant to their students and subjects. The teachers were from a range of curriculum areas, but predominantly science and health and physical education.

## Discussion

Drawing on the insights and expertise of teachers, these findings illuminate teacher priorities and perspectives when partnering with health researchers in health promotion education, in the context of sugary drinks. These understandings are important to better facilitate the integration of learning and research aimed at health behaviour change, given the limited time and demands on teachers and schools in crowded curricula where academic learning is a strong focus.

Internationally, a range of whole-school programmes and initiatives exist that mandate that teachers across all curriculum areas have a responsibility to contribute to supporting the health and well-being of students [35], [36–38]. An important factor influencing the facilitation and implementation of health promotion programmes is teacher perception and buy-in [13]. These are related to a range of factors including personal values and beliefs about the importance of health education and teachers’ roles in delivering this. In the New Zealand National Curriculum (NZC) Health and Physical Education curriculum learning area, health promotion is one of four underlying concepts that underpin health education knowledge [13, 24], and therefore it was expected that health teachers would see potential health learning benefits of the sugary drink context. What was noteworthy and encouraging was the investment that other teachers such as science teachers also felt about the health benefits of such learning. This aligns with the finding of Jourdan et al [39] that although it may not be a core part of their work, teachers feel strongly aligned to health promotion work because it resonates with their values and conviction that teaching and learning should extend beyond the classroom to contribute to wider societal well-being.

Teachers in the FIZZ curriculum study felt that through their collective action, they could positively influence health outcomes and their commitment to this learning extended to students’ families. While they were focused on immediate school-based goals such as assessment standards, this again demonstrates that teachers recognize and value the long-term benefits of health-related learning for the students and their communities. Importantly, teachers recognized students as health communicators in families and communities, as also acknowledged by Aghazadeh et al. [40].

Additionally, teachers in the FIZZ curriculum study indicated by their questions and comments a desire to extend their own professional learning. This finding aligns with that of Speller et al. [41] who found that teachers who received training in health promotion were more likely to be involved in health promotion activities in schools, indicating that teacher investment in health-based goals is influenced by training and personal competence.

In sum, these findings about learning for societal good and teacher learning about sugary drinks as a health-related context suggest motivations and rationale for why teachers might leverage curriculum decision-making towards student understanding of health behaviour change within already-crowded curriculum and school timetables.

Regarding learning transfer (the application of knowledge and skills acquired in one context to new contexts) and student action on learning, teachers play a pivotal role in facilitating this [42] by creating learning environments that encourage students to apply their knowledge to real-life health situations [43, 44]. Teachers in this study suggested multiple approaches that they thought would work for their students. They were interested in supporting students to initiate and carry out actions in a way that the students were given opportunities to experience success. This success was not so much oriented towards academic achievement but towards assisting them to begin to see and value themselves as young people who are able to help others and make a positive difference. This connects to Honneth’s [45] concept of esteem recognition within social justice theory, which emphasizes the significance for one’s identity and self-worth of being valued and recognized by others as having the ability to contribute positively to society. Further research is needed to understand how better to enhance the potential for operationalizing societal contribution and action, together with learning transfer-oriented conditions to support positive health behaviours (e.g. question decontextualization facilitating student reflections on decision-making more broadly and on health consequences for individual/family/community/national of different choices/behaviours) [46, 47]. Velasco et al. [47] argue that conditions required to facilitate learning transfer are influenced by implementation conditions: teaching methods, educational approaches and consistency with the broader school curriculum, further emphasizing the need for education–health codevelopment.

The drivers of health issues such as the overconsumption of sugary drinks are complex. Solutions that might mitigate the issue are entrenched in and shaped by the complex milieu of personal, familial, social, cultural, economic and other circumstances and thus rarely map directly onto individual behaviours and simplistic interventions. Therefore, even when adolescents care about their health and are aware of health risks associated with consuming sugary beverages, their behaviours are still influenced by these external drivers [48]. Recognizing this, teachers suggested multiple pathways for learning to achieve health goals within integrated curriculum, supporting student learning about the complexity of the environments in which sugary drinks are accessed and consumed. The socioecological perspective in the New Zealand H&PE curriculum supports understanding health promotion in terms of environmental influences and personal-level skills that support their own well-being and that of the community [24]. Students need to understand this situational complexity outside of the H&PE curriculum and across other learning areas, if they are to be given the tools to influence their own and others’ ‘in-the-moment’ decision-making and actions. Furthermore, students need to understand complexity in this way if, as contributing members of society, they are to prioritize and agitate for multilevel system changes [49]. For example, instead of attributing sugary drink overconsumption to a lack of individual willpower, students will be more likely to understand and support environmental changes such as reducing the influence of sugary drink producers and marketers and increasing public and political appetite for health-supporting policies such as a sugar tax.

Teachers stressed the importance of being able to apply their knowledge to make decisions about local curriculum in a way that engages their learners and ensures that learners experience the national curriculum in a way that is relevant to them, their families and the communities they live in. The findings of this study show that there may be little advantage in presenting ‘teacher-proof’ materials with an expectation of fidelity, because this will not happen if the materials are incompatible with any number of contextual influences like learner low literacy, and the materials will not be useful at scale [30]. A study by Campbell et al. [50] similarly revealed that teachers who amended lessons in a school-based diet and physical activity intervention did so to differentiate for student ability, update them for use with new technologies and enhance student engagement. On the other hand, variability in delivery and diversity within- and between-school contexts are suggested as possible reasons why evidence-informed school-based health education and health promotion practices do not always yield similar outcomes [18, 30, 51]. Some teachers will be less prepared or knowledgeable about the topic than others, and so the provision of background material including educative features for content knowledge and suggestions for pedagogical strategies is required, along with teacher professional learning, consultation and partnership for codevelopment of learning. These measures offer potential solutions to address evidence, suggesting that lack of connection to the core mission of the school is the most common reason for the failure of school-based health-promoting programmes [52]. Therefore, a ‘tight–loose’ balance must be achieved with materials designed and structured to support teacher adaptation while respecting the integrity and intent of the health-based (and other) learning objectives [26, 27, 53, 54]. One possible method for exploration with value-added proposition for teachers and students could be to explore assessment that orients students to action and to the societal value of learning [53], drawing on the push and focus within education literature for ‘learning that matters’ and using learning and assessment to orient students to goals and benefit outside of school-based credentialing and for society so wider than just the individual [55–58].

These findings about learning transfer, action and esteem recognition and teacher adaptation of curriculum materials demonstrate the importance of researchers working in collaboration with teachers who understand the curriculum and learners’ needs to design cross-curricular learning that balances school-based achievement goals with the development of health behaviours that matter.

## Conclusion

In conclusion, teachers from a range of curriculum areas see the value for students and society of school-based health promotion and strongly supported learning about sugary drinks as a relevant, adaptable context that appealed to both students and teachers. Consultation and codesign with teachers are key to ensure relevance and effectiveness. By better understanding school, teacher and student needs and aligning learning with curriculum and assessment goals, there is significant potential to increase the value proposition for teachers and students of learning for health promotion and behaviour change that has wider societal benefits.
